# Describing the learning curve of novices for the diagnosis of paediatric distal forearm fractures using point‐of‐care ultrasound

**DOI:** 10.1002/ajum.12291

**Published:** 2022-03-07

**Authors:** Peter J. Snelling, Philip Jones, Mark Moore, Peta Gimpel, Rosemary Rogers, Kong Liew, Robert S. Ware, Gerben Keijzers

**Affiliations:** ^1^ School of Medicine and Dentistry and Menzies Health Institute Queensland Griffith University Southport Queensland Australia; ^2^ 3555 Emergency Department Gold Coast University Hospital Southport Queensland Australia; ^3^ 67568 Sonography Innovation and Research (Sonar) Group Southport Queensland Australia; ^4^ Child Health Research Centre University of Queensland Southport Queensland Australia; ^5^ Emergency Department Queensland Children’s Hospital South Brisbane Queensland Australia; ^6^ Emergency and Trauma Centre Royal Brisbane and Women’s Hospital Herston Queensland Australia; ^7^ 3555 Faculty of Health Sciences and Medicine Bond University Gold Coast Queensland Australia

**Keywords:** competency, fractures, learning curve, musculoskeletal, nurse practitioner, paediatric distal forearm, point‐of‐care ultrasound, training

## Abstract

**Purpose:**

The learning curve of nurse practitioners (NPs) to accurately diagnose paediatric distal forearm fractures using point‐of‐care ultrasound (POCUS) was investigated.

**Methods:**

Each NP’s learning curve was calculated as cumulative diagnostic accuracy against a number of scans performed. The curve’s plateau represented the attainment of competency. Secondary outcomes were the comparisons before and after this cut‐off of diagnostic accuracy, classification of diagnostic errors, pain scores, duration and preference.

**Results:**

Five NPs performed 201 POCUS studies with diagnostic accuracy plateauing at 90%, providing a ‘cut‐off’ point at scan 15. Accuracy of POCUS scanning before and after the fifteenth scan was 81% (95% CI 70%–89%) and 90% (95% CI 84%–94%), respectively, demonstrating 9% improvement (P = 0.07). There was a 10% reduction in image interpretation errors. After fifteen scans, POCUS became faster (mean difference (MD) 2.6 min [95% CI 2.0–3.3], P < 0.001), less painful (MD 0.61 points FPSR scale [95% CI 0.04–1.18], P = 0.04) and more preferred by NPs (63% vs 77%, P = 0.03).

**Discussion:**

The learning curve of POCUS‐novice NPs independently scanning paediatric distal forearm injuries plateaued with mean diagnostic accuracy of 90% after 15 scans, suggesting competency was attained at this cut‐off, supported by higher accuracy, being faster, less painful and more preferred by NPs. Future training packages in forearm POCUS should further address image interpretation and provide ongoing expert feedback.

**Conclusions:**

The findings from this study suggest that competency in paediatric distal forearm POCUS can be attained by novices after a short training course and approximately 15 scans.

## Introduction

Forearm injuries account for almost 2% of all paediatric emergency department (ED) presentations.^1^ X‐rays are routinely ordered to diagnose distal forearm fractures.^2^ Approximately one‐third of these forearm injuries are diagnosed as buckle (torus) fractures of the distal radius metaphysis,^3^ for which a removable wrist splint rather than plaster immobilisation is the current standard of care in Australia.^4^

Point‐of‐care ultrasound (POCUS) is an alternative imaging modality to diagnose fractures in distal forearm injuries in children and can be conducted by a variety of users including ED physicians and nurse practitioners (NPs).^5^, ^6^ POCUS can be used to detect and differentiate clinically important fractures, which could potentially negate the requirement for X‐ray imaging in patients with either no fracture or a buckle fracture.^3^, ^5^ Additional benefits of POCUS are that it is well tolerated and preferred by parents.^3^ However, it has not yet been established how much practice is required for POCUS novices to become accurate at diagnosing paediatric distal forearm fractures.

The standard POCUS protocol for forearm injuries involves scanning six views of the forearm, whereby the distal radius and ulna are interrogated on their dorsal, lateral and volar aspects with a high‐frequency linear probe in a longitudinal axis with the probe marker orientated distally.^3^ Studies investigating this technique, or similar, have trained a variety of users for durations ranging from 30 min to 2 h.^3^, ^7^, ^8^, ^9^, ^10^, ^11^ However, it is not known how many scans are required to achieve sustained acceptable diagnostic accuracy.

The purpose of this study was to describe the learning curve of NPs, who were true novices to POCUS, to accurately diagnose distal forearm fractures using POCUS after limited training. The primary aim was to determine how many scans were required before the learning effect diminished, using the plateau of the learning curve to represent the attainment of competency. Secondary aims included determining the classification of errors and examining whether patient‐centred outcomes, including pain, duration of POCUS and preference, differed before and after competency was attained.

## Methods and materials

### Study design and materials

This secondary analysis used data collected from a prospective diagnostic study.^3^ The main study demonstrated that NP‐administered POCUS had clinically acceptable diagnostic accuracy. It was conducted at Queensland Children’s Hospital, a large tertiary paediatric centre in Southeast Queensland, Australia, between February 2018 and April 2019.^3^

### Participants

Patients were eligible for the main study if they were aged 4–16 years, presented to the ED between 7 am and 10 pm, with an isolated, clinically non‐angulated distal forearm injury, which required further evaluation with X‐ray imaging, and an NP trained in forearm POCUS was available to scan. Exclusion criteria were injury older than 1 week at presentation; external imaging had already been performed; known bone disease, such as osteogenesis imperfecta; suspicion of non‐accidental injury; congenital bone malformation; open fracture; neurovascular compromise; and distracting injury or suspicion for another fracture (e.g. scaphoid or elbow).

### Nurse practitioners

NPs are utilised in the ambulatory care area of the ED where they provide high‐quality, cost‐effective care.^12^, ^13^ NP scope of practice is broader than other traditional nursing roles and includes prescribing medications, initiating diagnostic imaging and laboratory tests, referring to specialists and admitting and discharging patients.^14^ NPs are primary care providers in many rural and remote healthcare facilities.^15^ Many children presenting to EDs with forearm injuries are reviewed primarily by NPs.

### Interventions

**Figure 1:**
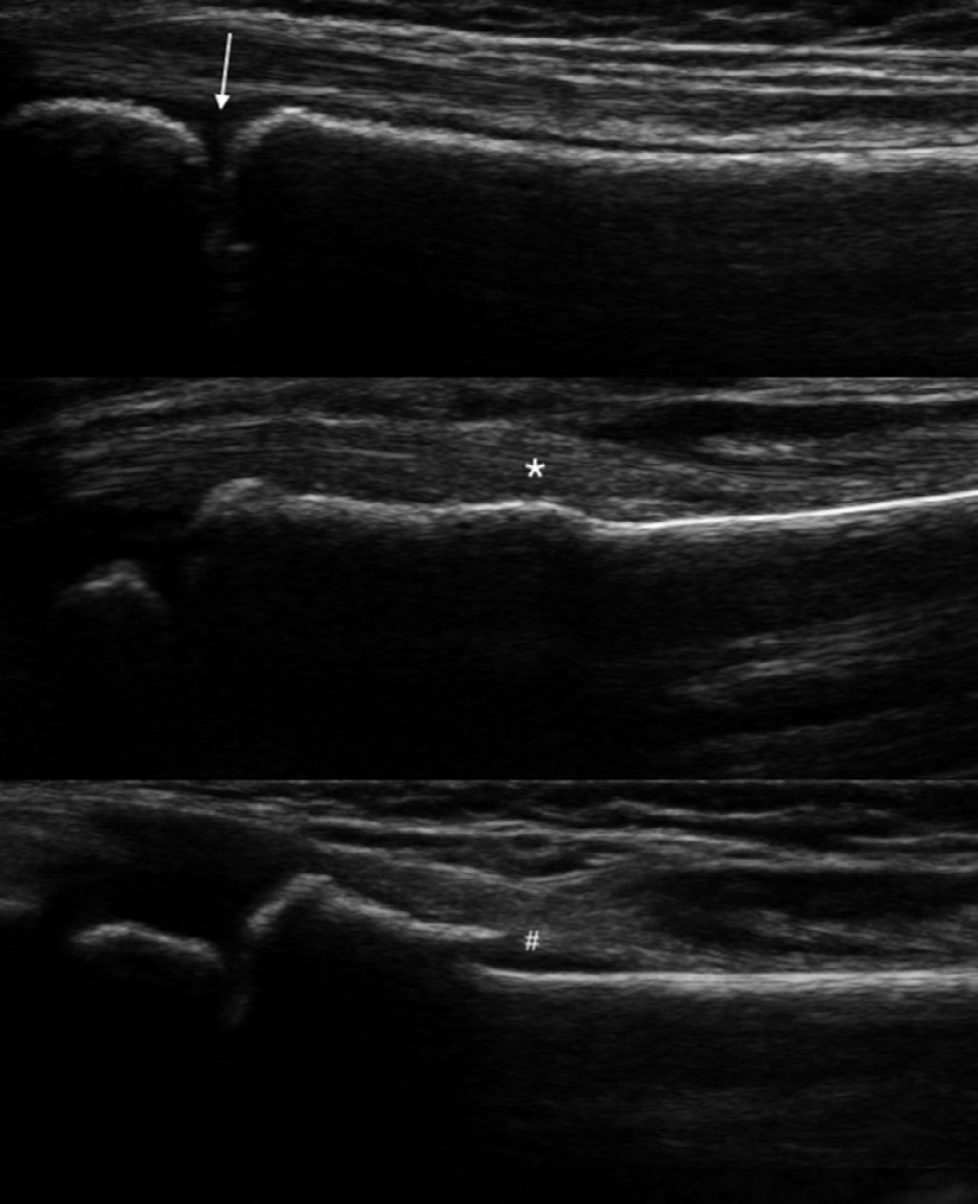
Distal radius ultrasound images demonstrating the different forearm injury categories: no fracture (arrow pointing to physis), buckle fracture (*) and cortical breach fracture (#).

Six NPs, with no prior POCUS experience, underwent a 2‐hour POCUS training course, which consisted of a staged learning package with lectures (including ultrasound imaging examples of normal bones and the different fracture types), progressing to practical training on each other’s arms and simulated models of paediatric distal forearm fractures,^16^ followed by 3 proctored scans on patients. All enrolled participants received both POCUS (HFL50xp/15‐6MHz, Fujifilm Sonosite Xporte, Bothell, Washington, USA) and X‐ray. When rostered and available on shift, NPs prospectively scanned eligible patients using the 6‐view POCUS protocol and classified the overall forearm (radius and ulna fractures considered) as ‘no’, ‘buckle’ or ‘other’ fracture, labelling their final image to prospectively document their diagnosis (Figure 1). This was then compared against the reference standard of a 2‐view X‐ray reported by a paediatric radiologist who was blinded to POCUS findings. A ‘buckle’ fracture was defined by an inward or outward bulge of the bone cortex without cortical breach on any aspect. ‘Other’ fractures were broadly defined as having a cortical breach, which included greenstick, complete or Salter‐Harris (physeal) types,^17^ but also included fractures at other sites (e.g. proximal forearm). At study completion, all POCUS images were reviewed by a POCUS expert, a paediatric emergency medicine physician with POCUS fellowship training, masked to both the NP diagnosis and X‐ray imaging.

### Outcome measures

A scan was defined as correct when the identified fracture category using POCUS matched the category identified using X‐ray imaging. This learning curve study evaluated diagnostic accuracy by NP experience by grouping NP‐administered POCUS scans by the cumulative number of scans completed by the performing NP. The primary outcome was the number of scans at which the learning curve plateaued (the cut‐off) in diagnostic accuracy, representing the attainment of competency.

Secondary outcome measures included the diagnostic accuracy of NP‐administered POCUS before and after the attainment of competency, as well as the diagnostic accuracy of ‘buckle’ and ‘other’ fractures, classification of diagnostic errors, pain score during POCUS (patient‐reported and parent observed), duration of POCUS imaging and preference (patient, parent and NP). Incorrect POCUS diagnoses, according to the reference standard in the parent study, were classified into the categories of ‘clinical indication’, ‘image acquisition’, ‘image interpretation’ or ‘reference standard’ error. Pain was assessed using the Faces Pain Scale‐Revised (FPSR), with patient and parent independently selecting one of six faces corresponding to maximum pain experienced (score 0–10).^18^ Duration of POCUS was recorded with a digital timer, which included the time taken to locate and turn on the machine, enter patient details and save the final image. Preference for POCUS was recorded as a binary variable for NPs and independently using a 5‐point Likert‐type scale for patients and parents.^3^

### Data analysis

Data were analysed for NPs who independently performed scans on 10 or more patients, as this was considered the minimum for potentially achieving a plateau in the learning curve.^19^ For these NPs, completed POCUS scans were ordered by the number of scans previously performed by that NP. The proportion of patients correctly diagnosed with NP‐administered POCUS compared to the reference standard of X‐ray was calculated following collating consecutive scans into blocks of 10. Diagnostic accuracy at each number of scans was defined by the diagnostic accuracy of all NPs over the next 10 completed scans. For example, the diagnostic accuracy at scan 15 was the diagnostic accuracy of all NPs over their 16^th^ to 25^th^ completed scans. The number of scans required to represent the attainment of competency was defined by the number of completed scans at which prospective diagnostic accuracy initially plateaued on the learning curve.

Summary statistics were described using frequency and percentage for categorical data and using mean and standard deviation (SD) or median and interquartile range (IQR) for continuous data as appropriate. Diagnostic accuracy, sensitivity and specificity were reported as percentages with corresponding 95% confidence intervals (CIs) calculated using the Wilson method. Hypothesis testing was performed using the chi‐squared test for independence for categorical data and two proportion Z‐test for difference in proportions. Pain scores and POCUS duration for scans performed before and after the learning curve plateau were compared using Student’s *t*‐test. Patient/parent preference were evaluated using the Wilcoxon signed rank test. All statistical calculations were performed using Stata/IC v14 (StataCorp, College Station, TX, USA).

## Ethics approval

The Children’s Health Queensland Hospital and Health Service Human Research Ethics Committee approved the study (HREC/17/QRCH/239). Written consent was obtained for all patients from their legal guardians prior to enrolment. NPs consented to being involved in this study.

## Results

**Figure 2:**
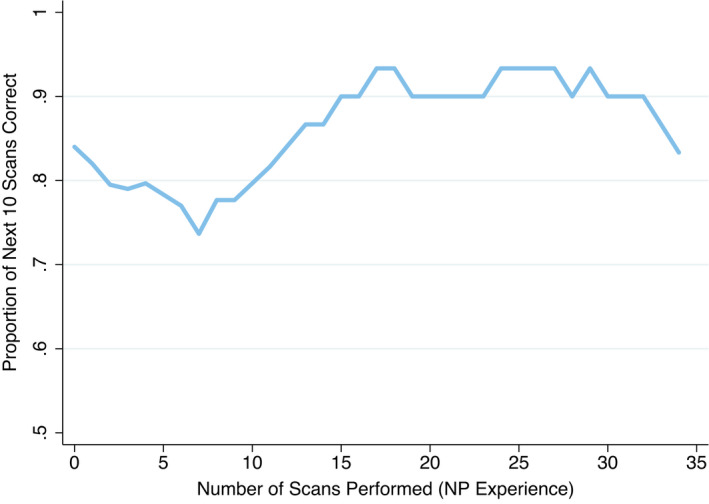
Diagnostic accuracy by NP experience, plotted as percentage correct over next 10 scans of each NP.

Two hundred and four patients were consecutively recruited in the main study, with 129 (63%) having a diagnosis of ‘buckle’ or ‘other’ fracture according to X‐ray. Six NPs with no previous POCUS experience were trained, with five performing 10 or more scans for a total of 201 POCUS studies (Table 1). Following the collation of consecutive scans into 10 scan blocks by each NP, the diagnostic accuracy of these NPs was consistently 90% from the 21 to 30 scans block onwards (Table 2). The combined diagnostic accuracy plateaued at 90%, providing a ‘cut‐off’ point at scan 15 (Figure 2). Age, sex, analgesia received, triage category and final diagnosis by X‐ray imaging were similar before and after the fifteenth scan (Table S1).

**Table 1:** Results by nurse practitioner (all participating).

Nurse practitioner (de‐identified)	Total scans	Proportion correct scans	Proportion correct—first 15 scans	Proportion correct—later scans
1	84	0.89 (75/84)	0.87 (13/15)	0.90 (62/69)
2	49	0.86 (42/49)	0.73 (11/15)	0.91 (31/34)
3	44	0.86 (38/44)	0.80 (12/15)	0.90 (26/29)
4	13	0.77 (10/13)	0.77 (10/13)	–
5	11	0.91 (10/11)	0.91 (10/11)	–
6	3	0.67 (2/3)	0.67 (2/3)	–

**Table 2:** Test performance characteristics of POCUS compared to reference standard of X‐ray imaging, stratified by experience of NP performing scan (n = 201).

Diagnostic study	Number of scans	Correct diagnosis	Proportion correct (95% CI)	No fracture	Buckle fracture	Other fracture
By group of 10 scans						
Scan 1–10	50	42	0.84 (0.71–0.92)	19/21 (0.90)	15/18 (0.83)	8/11 (0.73)
Scan 11–20	34	27	0.79 (0.63–0.90)	6/9 (0.67)	9/11 (0.82)	12/14 (0.86)
Scan 21–30	30	27	0.90 (0.74–0.97)	10/12 (0.83)	10/10 (1.00)	7/8 (0.88)
Scan 31–40	30	27	0.90 (0.74–0.97)	10/11 (0.91)	9/9 (1.00)	8/10 (0.80)
Scan 41–50	23	21	0.91 (0.73–0.98)	7/8 (0.88)	8/8 (1.00)	6/7 (0.86)
Scan 50+	34	31	0.91 (0.77–0.97)	11/13 (0.85)	15/15 (1.00)	5/6 (0.83)
First 15 scans vs later scans						
First 15 Scans	69	56	0.81* (0.70–0.89)	21/26 (0.81)	22/26 (0.85**)	13/17 (0.76)
Later than 15^th^ Scan	132	119	0.90* (0.84–0.94)	42/48 (0.88)	44/45 (0.98**)	33/39 (0.85)

POCUS, point‐of‐care ultrasound; NP, nurse practitioner.

Test performance reported as proportion (95% confidence interval calculated with Wilson method).

Fracture subtypes reported as number correct/number with final diagnosis (proportion correct).

*P = 0.071 for the chi‐squared test of independence.

**P = 0.037 for the chi‐squared test of independence.

Accuracy of POCUS scanning performed after the fifteenth scan was 90% (95% CI 84%–94%), with correct diagnosis achieved in 119/132 scans (Table 2). Accuracy prior to scan 15 was 81% (95% CI 70%–89%), with an observed improvement in accuracy of 9% (P = 0.07). Diagnostic accuracy for ‘buckle’ fractures was near perfect after attaining competency, with 44/45 diagnosed correctly (98%) compared to 22/26 prior to this cut‐off (85%). Improvements in diagnostic accuracy before and after the fifteenth scan were seen for patients with ‘no’ (81% first 15 scans vs 88% later scans) or ‘other’ (76% first 15 scans vs 85% later scans) fractures (Tables 2, S2).

Image interpretation errors occurred frequently prior to scan 15 but were reduced afterwards by 10% (Table 3). After the fifteenth scan, the proportion of errors due to image acquisition marginally increased. A recurrent missed clinically relevant finding was a cortical breach of the volar aspect of the distal radius with a coexistent buckle fracture of the dorsal aspect. Although reducing in proportion, normal findings were still misinterpreted as pathology after the cut‐off. Diagnostic errors were also observed in clinical assessment (patients included with proximal rather than distal radius fractures) and the reference standard (true injuries not visualised on X‐ray or false‐positive X‐ray findings).

**Table 3:** Classification of diagnostic errors for first 15 scans compared with later scans.

	Classification of diagnostic errors				
	Image interpretation^a^ n (%)	Image acquisition^b^ n (%)	Clinical indication^c^ n (%)	Reference standard^c^ n (%)	Total
First 15 scans (n = 69)	10 (14.5%)	1 (1.4%)	1 (1.4%)	1 (1.4%)	13 (18.8%)
Later scans (n = 132)	6 (4.5%)	4 (3.0%)	2 (1.5%)	1 (0.8%)	13 (9.8%)
Total	16	5	3	2	26

Results shown as number (percentage of all scans in subgroup).

^a^ First 15 scans—5 over‐called normal studies, 2 over‐called buckle fracture as cortical breach, 2 missed buckle fracture and 1 under‐called ulnar styloid fracture. Later scans—3 over‐called normal studies, 2 missed cortical breach fractures and 1 distal radius buckle over‐called as cortical breach.

^b^ First 15 scans—1 missed volar radius cortical breach. Later scans—3 missed volar radius cortical breach and 1 unclear cortical view overcalled as abnormal.

^c^ Clinical Indication Error—proximal or mid forearm fractures. Reference Standard Error—false‐positive or false‐negative X‐ray findings.

After scan 15, NPs were faster at scanning (mean difference (MD) 2.6 min (95% CI 2.0–3.3, P < 0.001)), (Table 4); an improvement was observed in child reported pain scores (MD 0.61 points FPSR scale [95% CI 0.04–1.18], P = 0.04); and NPs showed an increased preference for POCUS over X‐ray, 63% (42/67) vs 77% (102/132) P = 0.03. Responses from children and parents for preference for POCUS or X‐ray were similar for early versus later scans (Table S3).

**Table 4:** Differences in POCUS duration, pain score, proportion with severe pain and NP preference for first 15 scans compared to later scans (n = 201).

	First 15 scans (n = 69)	Later scans (n = 132)	Difference (95% CI)	P‐value
POCUS duration—mean (95% CI)				
Time (Minutes)	9.44 (8.68–10.2)	6.82 (6.55–7.09)	2.62 (1.97–3.27)	<0.001
FPSR score—mean (95% CI)				
Child	1.94 (1.42 – 2.47)	1.33 (1.02 – 1.65)	0.61 (0.04–1.18)	0.04
Parent	1.34 (0.95–1.73)	1.13 (0.86–1.39)	0.21 (−0.25 to 0.67)	0.37
Proportion with FPSR score ≥ 6^a^				
Child	0.10 (7/69)	0.04 (5/132)	0.06 (−0.01 to 0.14)	0.07
Parent	0.01 (1/68)	0.02 (2/132)	0.0 (−0.04 to 0.03)	0.98
NP preference—Proportion of scans^a^				
Prefer POCUS over XR	0.63 (42/67)	0.77 (102/132)	−0.15 (−0.28 to −0.01)	**0.03**

FSPR, faces pain scale‐revised; IQR, interquartile range; POCUS, point‐of‐care ultrasound; NP, nurse practitioner.

Pain scores not obtained from 1 parent in first 20 scans group. NP preference not recorded following 2 scans in first 20 scans group.

^a^ Chi‐squared significance level and Wald 95% confidence interval reported.

## Discussion

When novice POCUS clinicians scanned children with a clinically non‐angulated distal forearm injury after a short training course, diagnostic accuracy plateaued at approximately 90% after performing 15 scans, suggesting this was the point at which competency was attained. Competency was also supported at this cut‐off by observing an increase in diagnostic accuracy (particularly for buckle fractures), decreased duration of performing POCUS, reduced child pain scores and increased NP preference.

POCUS is a skill that can be broken down into five key areas: (1) indications for the scan; (2) knowledge of the anatomy being scanned; (3) acquiring the images; (4) interpretation of the images; and (5) relating the findings back to patient care.^20^, ^21^, ^22^ POCUS for a particular modality should be taught with each of these components addressed and then integrated. Once POCUS has been learnt for one application, it innately becomes easier to adapt the skills to additional applications.^19^ In particular, image acquisition requires visual perception and motor coordination of the probe, which may be the most challenging component of POCUS for true novices.

Diagnostic accuracy was used as the main indicator for the assessment of forearm POCUS competency, which provided an overall assessment that encompassed the entire composition of the skill. However, this measure does not discriminate false positives and false negatives and does not identify which component of the above five key areas accounted for an incorrect diagnosis. The attainment of competency at the cut‐off of 15 scans was supported by secondary outcomes including pain, duration and NP preference, which are patient‐centred and may reflect improved dexterity of technique, speed and acceptability. Others have studied the determination of acceptable performance using a validated assessment tool,^23^ comparison to a reference standard, comparison to expert findings^20^, ^24^, ^25^ or use of a validated training model.^26^ Furthermore, some have studied image interpretation alone.^27^, ^28^, ^29^

The training for the NPs in our study was comparable to other studies using a 6‐view distal forearm POCUS protocol for paediatric patients. However, in these other studies ED clinicians were already trained in other POCUS modalities or were already considered expert POCUS users.^7^, ^9^, ^10^, ^11^ In one study, after a logbook of 25 scans, ED physicians with greater than 2 years of POCUS experience were able to diagnose distal forearm fractures, compared to X‐ray reference standard, with a sensitivity of 94.7% and a specificity of 93.5%.^10^ In another study involving orthopaedic clinicians with significant previous ultrasound experience, the learning curve for distal forearm scanning proficiency was estimated at around 50 scans to attain a diagnostic accuracy of 98%. However, they were able to consistently scan at more than 90% accuracy after 10 patients.^8^

The plateau at 90% diagnostic accuracy is likely to be the ‘ceiling’ at which novices could independently attain after the training package, without ongoing expert feedback. Given the lack of expert feedback, NP improvement during the study could only be achieved by independent self‐reflection, with comparison of their POCUS findings to X‐ray findings, with the identification of their error and future adjustment, which may have been limited on a busy shift. This need for adequate training and regular expert feedback was highlighted by a recurrence of diagnostic errors due to either inadequate image acquisition or incorrect image interpretation. Normal findings were a source of image interpretation errors, although this improved over time. Additionally, the most frequent image acquisition error, leading to a missed clinically relevant fracture, was a cortical breach of the volar aspect of the distal radius in the presence of concurrent buckling of the dorsal radius (an image acquisition error likely related to confirmation bias). Future training should have an emphasis on these issues, which could include an image bank of normal and pathological findings. Ideally, this training should be supplemented with spaced repetition and regular expert feedback^30^ during the attainment of a logbook of at least 15 scans.

Although the observed improvement in diagnostic accuracy of 9% after the cut‐off of 15 scans was considered clinically significant, our study was not powered to statistically confirm this. The diagnostic accuracy improved after the cut‐off for buckle fractures, supporting the utility of POCUS for this particular entity with possible avoidance of X‐rays.^31^ Furthermore, the diagnostic accuracy of cortical breach fractures using POCUS could potentially be further enhanced by incorporating secondary signs, such as the pronator quadratus hematoma sign.^32^

Several emergency medicine professional society guidelines recommend that ED trainees should complete a benchmark of 10–50 quality‐reviewed examinations in any particular application but that the learning curve is dependent on the experience of the practitioner and the modality.^19^, ^33^, ^34^ Based on our study, true POCUS novices were able to attain competency in paediatric distal forearm scanning after three proctored scans and an additional 15 scans performed independently.

Strengths of this study include the novel description of the POCUS learning curve for true novices, compared to other studies, and the determination of competency based on diagnostic accuracy with the support of patient‐centred outcomes. Limitations of this study included it being a retrospective analysis of a prospective diagnostic single‐centre study. The sample size was limited, with an unequal distribution of scans before and after the cut‐off, which may have affected the analysis. Only 3 NPs completed 15 scans to achieve competency in this study, and none of these attained greater than 95% diagnostic accuracy. Although providing valuable insights into the learning curve for forearm POCUS, future studies should validate this approach with a broader range of clinicians to increase its generalisability.

## Conclusion

The learning curve of POCUS‐novice NPs, when independently scanning clinically non‐angulated paediatric distal forearm injuries after a short training course (including three proctored scans), reached a plateau in mean diagnostic accuracy of 90% after 15 scans, suggesting the cut‐off for the attainment of competency. Future training packages in forearm POCUS should include ongoing expert feedback and consider the inclusion of an image bank of normal and pathological findings, as image interpretation was a common source of error.

## Authorship statement

We confirm that (i) the authorship listing conforms to the journal’s authorship policy and (ii) that all authors agree with the content of the submitted manuscript.

## Funding

No funding information is provided.

## Conflict of interest

No conflict of interest to declare.

## Author contributions

**Peter James Snelling:** Conceptualization (equal); Data curation (equal); Methodology (lead); Project administration (lead); Resources (lead); Writing – original draft (lead). **Philip Jones:** Conceptualization (supporting); Data curation (equal); Formal analysis (lead); Methodology (equal); Project administration (supporting); Software (equal); Writing – review & editing (equal). **Mark Moore:** Data curation (equal); Writing – review & editing (supporting). **Peta Gimpel:** Data curation (equal); Writing – review & editing (supporting). **Rosemary Rogers:** Data curation (equal); Writing – review & editing (supporting). **Kong Liew:** Project administration (supporting); Resources (supporting); Writing – review & editing (supporting). **Robert Ware:** Conceptualization (equal); Formal analysis (equal); Methodology (equal); Project administration (equal); Supervision (equal); Writing – review & editing (lead). **Gerben Keijzers:** Conceptualization (equal); Formal analysis (equal); Methodology (equal); Project administration (equal); Supervision (lead); Writing – review & editing (lead).

## Supporting information


**Table S1** Patient characteristics per first 15 scans of NP compared to later scans (n = 201).
**Table S2** 3 × 3 diagnostic tables for first 15 scans and later scans.
**Table S3** Child and parent preference for POCUS over X‐ray by NP experience.
