# Supplementary material for: Describing the learning curve of novices for the diagnosis of paediatric distal forearm fractures using point‐of‐care ultrasound
Source: Australas J Ultrasound Med. 2022 Mar 7;25(2):66–73. doi: 10.1002/ajum.12291 (PMC9201201; doi:10.1002/ajum.12291)
Supplement: Supplementary file 1 — Table S1 Patient characteristics per first 15 scans of NP compared to later scans (n = 201). Table S2 3 × 3 diagnostic tables for first 15 scans and later scans. Table S3 Child and parent preference for POCUS over X‐ray by NP experience. [file AJUM-25-66-s001.docx]

**SUPPORTING INFORMATION**

**Table S1**. Patient characteristics per first 15 scans of NP compared to later scans ($n=201$)

| **Characteristic** | **First 15 Scans**  ($n=69$) | **Later Scans**  ($n=132$) |
| --- | --- | --- |
| Age (y) | 9.5 (±3.1) | 9.5 (±3.0) |
| **Gender** | | |
| Male | 38 (55) | 68 (52) |
| Female | 31 (45) | 64 (48) |
| **Side** | | |
| Right Arm | 32 (46) | 45 (34) |
| Left Arm | 37 (54) | 87 (66) |
| **Analgesia Received** | | |
| No Analgesia | 16 (23) | 27 (20) |
| Paracetamol and/or Ibuprofen | 52 (75) | 98 (74) |
| Opioid Analgesia | 1 (1) | 7 (5) |
| **Triage Category** | | |
| 3 | 8 (12) | 21 (16) |
| 4 | 51 (74) | 98 (74) |
| 5 | 10 (14) | 13 (10) |
| **Final Diagnosis** | | |
| No Fracture ($n=75$) | 26 (38) | 48 (36) |
| Buckle Fracture ($n=71)$ | 26 (38) | 45 (34) |
| Other Fracture ($n=$ 58) | 17 (25) | 39 (30) |
| Results shown as mean ($\pm$standard deviation) or number (% of subgroup).  NP = Nurse Practitioner | | |

**Table S2.** 3x3 diagnostic tables for first 15 scans and later scans

| **First 15 Scans** | **X-ray Diagnosis** | | |  |
| --- | --- | --- | --- | --- |
| **POCUS Diagnosis** | Other Fracture | Buckle Fracture | No Fracture | Total |
| Other Fracture | 13 | 2 | 0 | 15 |
| Buckle Fracture | 3 | 22 | 5 | 30 |
| No Fracture | 1 | 2 | 21 | 24 |
| Total | 17 | 26 | 26 | 69 |

| **Later than 15 Scans** | **X-ray Diagnosis** | | |  |
| --- | --- | --- | --- | --- |
| **POCUS Diagnosis** | Other Fracture | Buckle Fracture | No Fracture | Total |
| Other Fracture | 33 | 1 | 3 | 37 |
| Buckle Fracture | 3 | 44 | 3 | 50 |
| No Fracture | 3 | 0 | 42 | 45 |
| Total | 39 | 45 | 48 | 132 |

**Table S3.** Child and parent preference for POCUS over x-ray by NP experience

|  | **Likert Scale^†^ Response Frequency** | | | | | | |
| --- | --- | --- | --- | --- | --- | --- | --- |
| Diagnostic Study | Strongly Agree | Agree | Neither | Disagree | Strongly Disagree | Total | *P*-value* |
| **Child** | | | | | | | |
| First 15 Scans | 10 | 22 | 22 | 12 | 2 | 68 | 0.71 |
| Later than 15 Scans | 10 | 50 | 45 | 22 | 3 | 130 | -- |
| **Parent** | | | | | | | |
| First 15 Scans | 13 | 25 | 24 | 5 | 1 | 68 | 0.39 |
| Later than 15 Scans | 21 | 63 | 41 | 5 | 0 | 130 | -- |
| **†** “You prefer the use of ultrasound compared with x-ray imaging for the detection of your forearm fracture” for children and “You prefer the use of ultrasound compared with x-ray imaging for the detection of your child’s forearm fracture” for carers.  * *P*-value for Wilcoxon rank-sum test.  POCUS = Point-of-care Ultrasound; NP = Nurse Practitioner | | | | | | | |
